# High prevalence of asymptomatic *Plasmodium* infection in Bandafassi, South-East Senegal

**DOI:** 10.1186/s12936-021-03746-7

**Published:** 2021-05-12

**Authors:** Aida Sadikh Badiane, Tolla Ndiaye, Alphonse Birane Thiaw, Deme Awa Binta, Mamadou Alpha Diallo, Mame Cheikh Seck, Khadim Diongue, Mamane Nassirou Garba, Mouhamadou Ndiaye, Daouda Ndiaye

**Affiliations:** 1 Laboratory of Parasitology and Mycology, Cheikh Anta Diop University of Dakar, Dakar, Senegal; 2 Laboratory of Parasitology and Mycology, Aristide Le Dantec Hospital, Dakar, Senegal

**Keywords:** Asymptomatic malaria, *Plasmodium* species, Senegal

## Abstract

**Background:**

Malaria control and elimination strategies are based on levels of transmission that are usually determined by data collected from health facilities. In endemic areas, asymptomatic *Plasmodium* infection is thought to represent the majority of infections, though they are not diagnosed nor treated. Therefore, there might be an underestimation of the malaria reservoir, resulting in inadequate control strategies. In addition, these untreated asymptomatic *Plasmodium* infections maintain transmission, making it difficult or impossible to reach malaria elimination goals. Thus, the aim of this study was to determine the prevalence of asymptomatic *Plasmodium* infections in southeastern Senegal.

**Methods:**

A cross sectional study was conducted among asymptomatic individuals (N = 122) living in the village of Andiel located in Bandafassi, Kédougou, which consisted of about 200 inhabitants during the malaria transmission season in late October 2019. For each individual without malaria-related symptoms and who consented to participate, a rapid diagnostic test (RDT) was performed in the field. Results were confirmed in the laboratory with photo-induced electron transfer (PET-PCR).

**Results:**

Malaria prevalence was 70.3% by PET-PCR and 41.8% by RDT. During the same period, the health post of the area reported 49. 1% test positivity rate by RDT. The majority of the infected study population, 92.9%, was infected with a single species and 7.1% had two or three species of *Plasmodium*. *Plasmodium falciparum* was predominant and represented 90.2% of the infections, while 6.5% were due to *Plasmodium ovale* and 3.3% to *Plasmodium malariae*. 59.4% of children targeted for SMC (zero to ten years old) were infected.

**Conclusion:**

In southeastern Senegal, where the transmission is the highest, malaria control strategies should address asymptomatic *Plasmodium* infections at the community level. The results suggest that this area could be eligible for mass drug administration. Moreover, non-falciparum species could be more common and its prevalence should be determined countrywide.

## Background

Malaria is an endemic parasitic disease, with an estimated 229 million cases worldwide in 2020 [1], and most cases occurred in the World Health Organization (WHO) Africa region, with 225 million cases and 384,000 deaths. Although malaria burden declined in sub-Saharan African from 2000 to 2019, there is slower progress in both cases and deaths since 2014 [1]. In Senegal, malaria is endemic, with 354,708 reported symptomatic cases in 2019 in health facilities. Three regions in southeastern Senegal recorded 81% of all cases and 39% of the total mortality [2]. Malaria cases are reported in the health facilities among patients seeking care; however, many *Plasmodium* infections are not detected as in endemic areas individuals can carry the *Plasmodium* parasites without any symptoms. There is overwhelming evidence indicating that a significant proportion of *Plasmodium* infections are asymptomatic in endemic areas. It has been shown that over 25% of individuals in sub-Saharan Africa (Cameroon, The Gambia, Mali and Senegal) presented sub-microscopic gametocytes and were capable of infecting mosquitoes [3]. Likewise, in Laos, 20% (175/888) of the seemingly healthy individuals were found to be infected with *Plasmodium* species [4]. These asymptomatically infected persons thus do not seek treatment, remain infected, and serve as reservoirs for the duration of parasite carriage, maintaining malaria transmission [5].

Asymptomatic *Plasmodium* infections are a serious threat to elimination and should be addressed by the National Malaria Control Programmes in their policy implementation for malaria elimination. In endemic areas, the parasite density among subjects with asymptomatic *Plasmodium* infections is usually low, and generally not detected by classical methods, such as rapid diagnostic test (RDT) and light microscopy, which have low sensitivity compared to molecular techniques [6].

In Senegal, *Plasmodium falciparum* is the primary species responsible for malaria, but the prevalence of other species of *Plasmodium* remains unknown. However, in the context of malaria elimination, the prevalence of other *Plasmodium* species that may cause asymptomatic *Plasmodium* infections with low density infection or relapses must be determined. The circulation of non-falciparum species has been previously reported in Senegal; *Plasmodium ovale* and *Plasmodium malariae* have been found in Dakar and Kedougou [7–9], as well as the circulation of *Plasmodium vivax* in southeast Senegal [10]. However, data on non-falciparum species is sparse and the circulation of *P. vivax* is debated.

Asymptomatic *Plasmodium spp*. infections are usually not diagnosed as people are not likely to seek care in the absence of symptoms. In remote villages of high transmission zones, such as southeastern Senegal, asymptomatic *Plasmodium* infections may be more prevalent than symptomatic malaria cases detected in the health facilities. The aim of this study was to investigate the prevalence of asymptomatic *Plasmodium* infections in the village of Andiel in Kédougou, southeastern Senegal.

## Methods

### Study design

The study was a cross-sectional survey. In the village, after sensitization of the community by the local health team and community leaders, the inhabitants were asked if they were experiencing symptoms related to malaria; if the answer was negative, they were invited to participate in the study. Consenting participants who met the inclusion criteria received a malaria rapid diagnostic test using the *CareStart*™ Malaria Pf/Pan, (Accessbio, USA) and blood was collected from the finger prick and spotted on filter paper to create a dried blood spot (DBS), and stored at room temperature. All samples were tested with PET-PCR at the laboratory for confirmation and *Plasmodium* species identification. Non-consenting individuals or those with symptoms were referred to the HCP serving the village for a malaria rapid diagnostic test. All residents with a positive test received first line treatment according to the National Malaria Control Programme (NMCP) policy. Data from the health post of Bandafassi which oversees Andiel village was collected through the NMCP database. The study was approved by the ethical committee of the Ministry of Health of Senegal (00000171/MSAS/DPRS/CNERS).

### Setting

The study was conducted in the region of Kédougou. It is bordered on the west by the hills of Bassari country and Mount Assirik, which dominates the Niokolo-Koba National Park. Kédougou is one of the wettest regions in the country, with at least 1300 mm of rainfall/year. The rainy season lasts about six months, from May to October, followed by a long dry season. However, this rainfall is characterized by great spatio-temporal variability, the months of August and September being the rainiest [11]. Temperatures are generally high, with maximums ranging from 34 to 42 °C and minima from 21 to 25 °C. The study was conducted in the village of Andiel (12.5435895, − 12.3686038), the precinct (or borough) of Bandafassi, in the region of Kédougou in southeastern Senegal. Andiel is located 12 km from the health post of Bandafassi on which it depends and around 30 km from Kédougou, the district seat. There is no paved road to Andiel and no transport vehicles. The village is in a mountainous area, and while vehicles can access the Peulh village of Landièny at the bottom of the mountain, Andiel can only be reached on foot through the mountains. Because of this difficulty of access, visits to the health post are infrequent, and traditional medicine use is common. Andiel has around 200 inhabitants of the Bedik ethnicity who live in the mountains in small communities, preserving their ancestral way of life. Malaria is hyperendemic, with transmission starting in May–June up to December-January. Three Plasmodium species, *P. falciparum*, *P. ovale*, *P. malariae*, have been reported in the region and *P. falciparum* is the predominant species [7]. Since 2013, children in the region of Kédougou aged zero to ten years old have received seasonal malaria chemoprevention (SMC) from July to October (4 months). Home base management of malaria (HBMM) was started in the region since 2010 and the village of Andiel has one home care provider (HCP).

The study was carried out on apparently healthy participants in the village of Andiel during the malaria transmission season at the end of October 2019. Date of sampling and demographic data such as age and sex were collected for participants who were enrolled in the study. No information related to test result or epidemiological data were collected for non-consenting inhabitants and individuals with symptoms.

### Participants

All residents older than 6 months who did not present any malaria-related symptoms such as fever or history of fever, headaches, nauseas vomiting, aches and pains were eligible. Residents who presented symptoms consistent with malaria or reported being treated for malaria the last two weeks were ineligible.

### Laboratory investigation

The RDT used was the *CareStart*™ Malaria Pf/Pan, Accessbio. This two-band RDT detects the *P. falciparum* histidine rich protein 2 (PfHRP2) specific to *P. falciparum* and pan-*Plasmodium* parasite lactate dehydrogenase (p-LDH). This allows the RDT to distinguish *P. falciparum* from the other *Plasmodium* species in mono-infections or in mixed infections.

Molecular characterization of *Plasmodium* species was also performed for all samples using the photo-induced electron transfer (PET)-PCR assay [12] on a Roche LightCycler 96 instrument (Roche Molecular Systems, Inc). Each experimental run included both a negative (no template) and a positive (3D7 *P. falciparum* strain) control. Samples with a cycle threshold (CT) of 40 or less were scored as positive [12, 13]. The specificities of the *P. malariae*, *P. vivax*, and *P. ovale* was performed in a 20 ml reaction containing 2X TaqMan Environmental MasterMix 2.0 (Applied BioSystems), 250 nM each forward and reverse primer, and 5 μl of DNA template. All reactions were performed in a total volume of 20 μl containing: 5 μl of DNA, 10 μl of 2X ABI TaqMan buffer and 250 μM of each forward and reverse primers. The reactions were performed under the following cycling parameters: initial hot-start at 95 °C for 15 min, followed by 45 cycles of denaturation at 95 °C for 20 s and annealing at 60 °C for 40 s.

### Malaria incidence in the health post of Bandafassi

The health post of Bandafassi oversees twenty (20) villages, including Andiel, for a total of 7944 inhabitants. The number of malaria cases reported by the health post of Bandafassi which oversees Andiel village was obtained through the NMCP database (Data are aggregated, and not reported at the village level, thus data specific to Andiel were not available). Malaria cases were diagnosed among patients seeking care at the health post and who presented fever during the consultation or a history of fever during the preceding 48 h, using SD Bioline Malaria antigen Pf® RDT. The test positivity rate was calculated for all the patients attending the health post (general population) and for specific groups of patients (< 5 years old, children aged between 5 to 10 years old, patients older than 10 years excluding pregnant patients and pregnant women) during the malaria transmission season (from July to December).

### Statistical analysis

Data were collected into Excel and analyzed. Statistical analysis was performed using GraphPad prism version 5.01. Differences were considered significant with a p-value < 0.05. The non-parametric Mann–Whitney U test was used to calculate the p-value.

### Definitions

In this manuscript “symptomatic malaria cases” was used strictly for symptomatic malaria episodes, “asymptomatic *Plasmodium* infections” for infected individuals who did not present any symptoms and “Plasmodium infections” when referring to all infections (clinical and asymptomatic together). “Participants” was used for the individuals without malaria symptoms and who consented to participate in the study. Data were not collected from people with symptoms consistent with malaria. Asymptomatic *Plasmodium* infection was defined as the absence of fever or history of fever, headaches, nausea, vomiting, or aches and pains, with a positive biological malaria test (RDT or PCR).

## Results

### Demographics

**Fig. 1:**
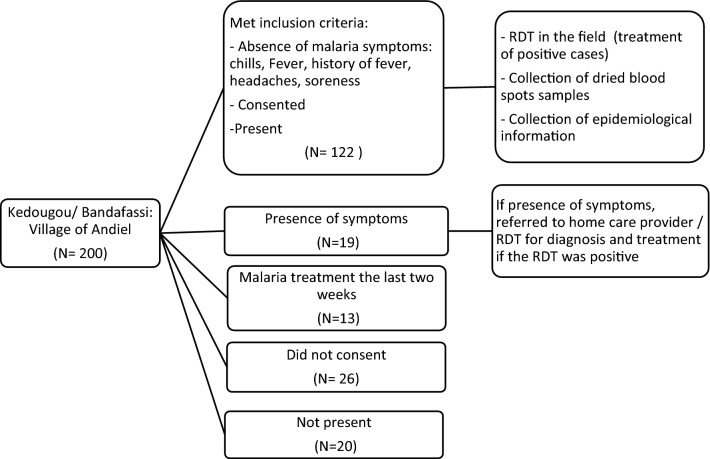
Flowchart of participant recruitment

The village of Andiel is composed of approximately 200 inhabitants, among whom one hundred twenty-two (122) met the inclusion criteria and were enrolled. Twenty-six (26) individuals did not present any malaria related symptoms, but did not consent for the study. Fifty-two (52) of the inhabitants did not meet the inclusion criteria: thirteen (13) reported malaria treatment during the two weeks before the study, nineteen (19) presented malaria related symptoms, and twenty (20) were not present during the period of the study (Fig. 1).

Among the 122 asymptomatic subjects included in this study, 54.9% (67) were female and 45.1% (55) were male. The participants' ages ranged from 11 months to 90 years with the mean (SD) of 27.2 (22.69) years and a median of 19 years (Q1–Q3: 10–43). Age distribution is summarized in Table 1. Children between 0 to 4 years old represented 9.8% (12), 5 to 9 years made up 14.8% (18); 10 to 14 years made up 17.2% (21) and individuals 15 years and older represented 58.2% (71).

**Table 1:** Demographic characteristics of the study population

Age group	Proportion of the study population by age group (%) (N = 122)	Sex	
		Female (%) (N = 67)	Male (%) (N = 55)
0–4 years	9.8 (12)	4.9 (06)	4.9 (06)
5–9 years	14.6 (18)	11.5 (14)	3.2 (04)
10–14 years	17.2 (21)	8.20 (10)	9 (11)
≥ 15 years	58.2 (71)	30.3 (37)	27.9 (34)

### Malaria prevalence

RDT was performed for all the 122 participants at the community level in the village; 121 samples were tested using PET-PCR in the laboratory (there was one missing sample). The prevalence of malaria was by PET-PCR was 70.2% (95% CI 29.2–53.9) and by RDT was 41.0% (95% CI 54.4–86.6).

### Evaluation of the diagnostic performance of the RDT

**Fig. 2:**
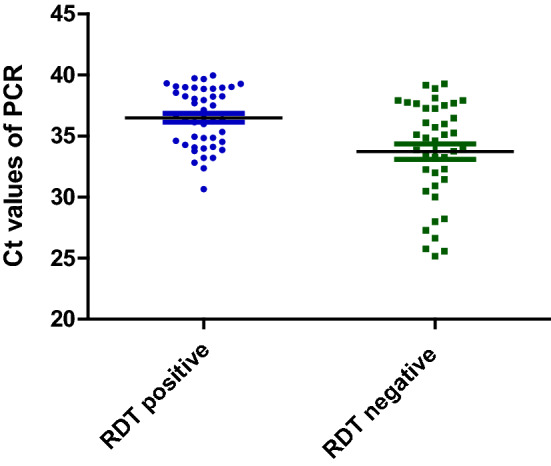
Ct comparison of the samples that were positive with PET-PCR and negative with RDT

The PET-PCR and RDT were both positive in 33.9% (41) of samples and both negative in 22.3% (27) of samples, while 7.4% (9) were positive by RDT and negative by PET-PCR, and 36.4% (44) were detected with the PET-PCR but not RDT. The Ct for the samples detected by PET-PCR but negative with the Pan/RDT were higher than the Ct for the samples positive for both techniques (Fig. 2), and the difference was statistically significant (Mann-Withney U, p- value = 0.001).

### Malaria detection and age

There was no statistical difference between age group detection tests (Mann–Whitney U, p-value = 0.07), though the sample size was small. Among the children aged 0 to ten years old (N = 32), who were targeted for SMC, 53.1% (N = 17) had asymptomatic *Plasmodium* infection by PET-PCR.

### *Plasmodium* species and mixed infections

*Plasmodium* species and prevalence of mixed infection were determined using PET-PCR. Among positive samples (85), single infections (caused by one *Plasmodium* species) represented 92.9% (77 *P. falciparum*, 1 *P. malariae*, 1 *P. ovale*), while 7.1% were mixed infections (5 *P. falciparum* + *P. ovale* and 1 *P. falciparum* + *P. malariae*). *Plasmodium vivax* was not detected.

The RDT identified *P. falciparum* bands in 94.1% (48/51) of the RDT positive samples, and *P. falciparum* and pan bands, suggesting the presence of non-falciparum species, in 5.9% (3/51 samples). Among the three samples with positive pan bands on RDT, two were mixed infections with *P. falciparum* and *P. ovale* and one was a *P. falciparum* single infection. Conversely, one *P. malariae* infection, one *P. ovale* infection, one mixed *P. falciparum* + *P. malariae* infection, and two mixed *P. falciparum* + *P. ovale* infection were not detected by RDT.

### Symptomatic confirmed malaria at the Bandafassi health post

**Fig. 3:**
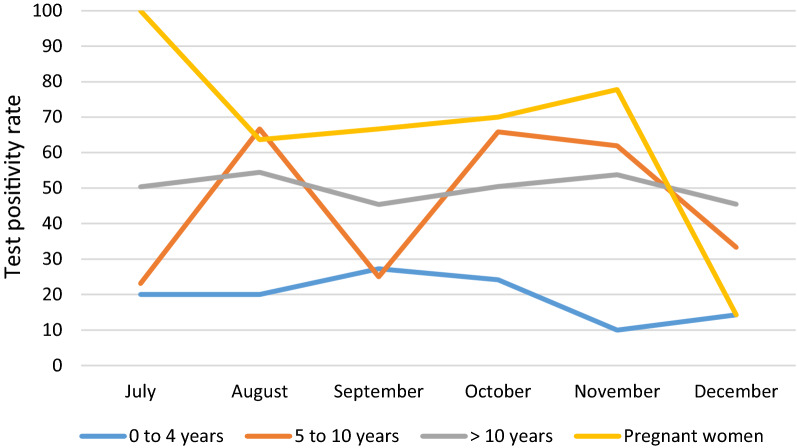
Malaria test positivity rate at Bandafassi health post by age group, July–December

The test positivity rate (TPR) at the health post of Bandafassi using RDT (SD Bioline Malaria antigen Pf® RDT) was determined during the transmission season at the same period (October) and was 40.7% (598/1468). While children 3 months to 10 years are targeted for SMC, the TPR from July to December 2019 among patients with febrile illness who attended the health post and received an RDT was lowest among children under five years old, while children aged 5 to 10 years had a TPR more comparable to older age groups during the same period (Fig. 3).

During the month of October in which the samples were collected in the village of Andiel, the TPR in the health facility of Bandafassi was 24.1%, 65.8%, 50.4%, respectively, among children under five, children aged 5 to 9 years and patients aged 10 or more years (excluding pregnant women, as this group was not represented in our participants) (Table 2).

**Table 2:** Comparison of malaria test positivity rate in the health post of Bandafassi and the village of Andiel during the month of October

Age	Bandafassi health post test positivity rate	Andiel RDT prevalence	Andiel PET-PCR prevalence
0–4 years	24.1% (7/29) 95% CI [3–13]	25.0% (3/12) 95% CI [1–7]	41.7% (5/12) 95% CI [2–9]
5–10 years	65.8% (27/41) 95% CI [20–33]	50.0% (10/20) 95% CI [5–15]	60.0% (12/20) CI [7–16]
> 10 years	50.4% (115/228) CI [100–130]	32.6% (30/92) 95% CI [21–40]	73.9% (68/92) 95% CI [59–76]

## Discussion

This community-based study conducted in southeast Senegal reports the prevalence of asymptomatic *Plasmodium* infection in a remote, high burden village. The results showed a prevalence of 70.3% by PET-PCR and 41.8% by Pan/RDT. During the same month, a test positivity rate (proportion of clinically ill patients who received a malaria test who tested positive) of 49.1% was recorded at the health post of Bandafassi using PfHRP2 RDT. The high Ct value of samples detected by PET-PCR but not by RDT (with a statistically significant difference) suggests that the parasite density is low for those samples. The low detection of asymptomatic *Plasmodium* infections by Pan/RDT compared to PET-PCR shows the importance of utilizing sensitive techniques such as real time PCR for malaria diagnosis during surveys.

Overall, 53.1% of children up to ten years old, the age group targeted for seasonal malaria chemoprevention, were infected with *Plasmodium* by PET-PCR. While the highest proportion and overall numbers of asymptomatic *Plasmodium* infection were detected by PET-PCR were in those 10 years and older, asymptomatic malaria infection remained startlingly high among those targeted for SMC, particularly among children 5–9 years old. Similarly, the test positivity rate at the health post was highest among children 5–9 years of age. This finding is concerning for the effectiveness of SMC not only in Andiel, but among children in this catchment zone, whether due to lower uptake, declining efficacy, or lower coverage of co-interventions, such as bed net use.

Although *P. falciparum* was the main species identified, *P. ovale* and *P. malariae* were detected mostly as mixed infections with *P. falciparum*, which have been reported in several regions of the country [7–10, 14] as well as in other African countries [15–17]. While, *P. falciparum* is predominant in sub-Saharan Africa, more attention should be given to the other species as their prevalence often becomes higher when the prevalence of *P. falciparum* decreases [18]. Moreover, the infections due the non-falciparum species are generally characterized by low parasite density and are usually asymptomatic. *Plasmodium vivax* was not identified in this study, however, some studies in Senegal have reported its presence in some parts of the country [7, 10]. In addition, a study has found serological markers (PvMSP1-19) of its circulation in the north and the centre of the country, but not in the South where this study was conducted [19]. More investigations are needed across the country to provide solid evidence of *P. vivax* circulation.

There were several important limitations to this study. It was conducted in a small village, and as such the findings are not representative and cannot be generalized. Non-inclusion of symptomatic malaria cases during the sampling at the community makes it impossible to directly compare to data from the health facility. It was also a missed opportunity to collect information about bed net availability and use and SMC coverage, which might have further illuminated the results.

However, this study gives important information about the frequency of asymptomatic *Plasmodium* infection in a high transmission setting, the discrepancy between PET-PCR and RDT-based parasite prevalence, which suggests that parasite prevalence may be higher in other regions of the country than currently thought. Molecular techniques such as PET-PCR should be integrated into cross sectional surveys in order to obtain a more accurate understanding of parasite prevalence and species differentiation.

## Conclusion

The high prevalence of asymptomatic malaria observed in this study suggests that malaria control strategies should address asymptomatic *Plasmodium* infections at the community level. Therefore, it becomes necessary to put in place a surveillance strategy for these infections that can serve as a reservoir for malaria transmission in a country such as Senegal, which is working towards malaria elimination in 2030. In Senegal, malaria surveillance became an intervention in low transmission settings, however it should be extended to the intermediate and high transmission areas such as Kédougou. The prevalence of non-falciparum species should be monitored nationally as these will become relatively more prevalent as *P. falciparum* decreases.

## Data Availability

The data supporting the findings of this article are included within the article.

## Abbreviations

ACT: Artemisinin-based combination therapy
CT: Cycle threshold
DBS: Dried blood spot
DSDOM: Dispensateur de soin à domicile
HCP: Home care provider
NMCP: National Malaria Control Programme
PECADOM: Prise en charge à domicile
PET-PCR: Photo-induced electron transfer PCR
PfHRP2: *P. falciparum* Histidine rich protein 2
p-LDH: Parasite lactate dehydrogenase
RDT: Rapid diagnosis test
SMC: Seasonal malaria chemoprevention
